# Obesity Correlates and Weight-Management Practices in a Clozapine-Treated Cohort: A Service Evaluation From Suffolk.

**DOI:** 10.1192/bjo.2026.11616

**Published:** 2026-06-30

**Authors:** Nada Zahreddine, Lucy Mountford, Edward Barton, Maria Giner-Murillo, Mohamed Elkoshiery, Angelos Zikos, Ahmed Ibrahim

**Affiliations:** Norfolk and Suffolk NHS Foundation Trust, Ipswich, United Kingdom; Angelos.zikos@nsft.nhs.uk, Ahmed.ibrahim@nsft.nhs.uk Both Core Psychiatry Trainees at Norfolk and Suffolk NHS Foundation Trust, Ipswich, UK]

## Abstract

**Aims:**

Clozapine is associated with increased risk of metabolic syndrome. Regular monitoring, lifestyle and pharmacological interventions are recommended. Service-level evaluation is required to identify suboptimal care and inform quality improvement.

**Methods:**

This cross-sectional service evaluation was conducted within a centralised clozapine clinic serving adult secondary care services across Suffolk. Clinical, physicalhealth, and weight-management data were extracted from electronic health records.

**Results:**

N=257; mean age 46.9 years (SD=12.4); 68.9% male; 35.2% smokers; 46.7% alcohol users (6.3% heavy drinkers); cannabis 0.8%; other drugs 1.6%; 33.7% reported constipation.

Mean body mass index (BMI) was 31.85 kg/m² (SD=7.05), with 54.5% meeting criteria for obesity and 29.2% classified as overweight. 12.6% met Hbs-540c criteria for prediabetes and 19.8% for type 2 diabetes (T2DM). 10.2% had elevated total cholesterol (>6.18 mmol/L), and 24.9% had blood pressure readings ≥140/90 mmHg. Clinician-recorded prevalences were 6.6% obesity, 9.7% prediabetes, 27.0% T2DM, 16.0% dyslipidaemia, 12.1% hypertension, and 5.5% cardiovascular disease.

Lifestyle advice was recorded for 22.7% of patients and pharmacological weight-management interventions for 7.1%. Although 14.8% of patients were prescribed adjunctive aripiprazole, this was documented specifically for weight management in only 10.5% of those. Metformin was prescribed in 25.2% of patients and was strongly associated with T2DM (p < .001). GLP-1 receptor agonists were used in 3.1% and were prescribed exclusively to patients with T2DM (p < .001). No patients were prescribed topiramate.

The multivariable linear regression model predicting BMI was statistically significant (p < .001). Higher BMI was independently associated with younger age, female gender, autism spectrum disorder/learning disability comorbidity, greater depressive symptom severity, higher systolic blood pressure, receipt of lifestyle advice or structured lifestyle intervention, and prescription of aripiprazole, valproate, or lamotrigine (all p ≤ .040).

There was marked discordance between BMI-defined obesity and clinician-recorded obesity or lifestyle advice (p < .001). Only 10% of patients meeting objective criteria for obesity had a documented obesity diagnosis, and only 29.7% had lifestyle advice recorded. Patients receiving lifestyle advice had significantly higher BMI than those who did not (p=.014), and those with a clinician-recorded obesity diagnosis also had higher mean BMI (although not significant). Together, these findings suggest that recognition and management of obesity occurred at higher BMI levels, rather than systematically once obesity criteria were met.

**Conclusion:**

Obesity was common but inconsistently recognised and managed in clozapine-treated patients, with evidence of threshold bias. Systematic identification and management of obesity is needed within clozapine services.

